# Synthesis and preliminary biological evaluation of gabactyzine, a benactyzine-GABA mutual prodrug, as an organophosphate antidote

**DOI:** 10.1038/s41598-022-23141-9

**Published:** 2022-10-27

**Authors:** Michal Weitman, Arik Eisenkraft, Zeev TaShma, Igor Makarovsky, David Last, Dianne Daniels, David Guez, Ran Shneor, Yael Mardor, Abraham Nudelman, Amir Krivoy

**Affiliations:** 1 Chemistry Department, Bar Ilan University, 52900 Ramat Gan, Israel; 2 The Institute for Research in Military Medicine, The Hebrew University Faculty of Medicine and The IDF Medical Corps, Jerusalem, Israel; 3 The IDF Medical Corps Headquarters, Ramat Gan, Israel; 4 The Advanced Technology Center, Sheba Medical Center, Ramat-Gan, Israel; 5 Sackler Faculty of Medicine, Tel-Aviv University, Tel-Aviv, Israel; 6 Geha Mental Health Center, Petach-Tikva, Israel

**Keywords:** Drug discovery, Medical research

## Abstract

Organophosphates (OPs) are inhibitors of acetylcholinesterase and have deleterious effects on the central nervous system. Clinical manifestations of OP poisoning include convulsions, which represent an underlying toxic neuro-pathological process, leading to permanent neuronal damage. This neurotoxicity is mediated through the cholinergic, GABAergic and glutamatergic (NMDA) systems. Pharmacological interventions in OP poisoning are designed to mitigate these specific neuro-pathological pathways, using anticholinergic drugs and GABAergic agents**.** Benactyzine is a combined anticholinergic, anti-NMDA compound. Based on previous development of novel GABA derivatives (such as prodrugs based on perphenazine for the treatment of schizophrenia and nortriptyline against neuropathic pain), we describe the synthesis and preliminary testing of a mutual prodrug ester of benactyzine and GABA. It is assumed that once the ester crosses the blood–brain-barrier it will undergo hydrolysis, releasing benactyzine and GABA, which are expected to act synergistically. The combined release of both compounds in the brain offers several advantages over the current OP poisoning treatment protocol: improved efficacy and safety profile (where the inhibitory properties of GABA are expected to counteract the anticholinergic cognitive adverse effects of benactyzine) and enhanced chemical stability compared to benactyzine alone. We present here preliminary results of animal studies, showing promising results with early gabactyzine administration.

## Introduction

Organophosphates (OPs) are powerful inhibitors of acetylcholinesterase (AChE), with a deleterious central nervous system (CNS) effect^1,2^. As a consequence of this inhibition, a progression of toxic signs is evident, including hypersecretion, tremors, convulsions, respiratory distress, and, ultimately, death^3,4^. The initial phase is the result of massive acetylcholine (ACh) accumulation leading to excessive cholinergic activity^5,6^. Thus, the primary targets of medical countermeasures (MCM) against OP poisoning are to prevent further AChE inhibition and reactivation and counteracting the effects of ACh in muscarinic receptors^7^. However, while present MCMs for OP poisoning offer robust protection against lethality^7,8^, CNS damage, including long-lasting behavioral deficits, are not completely prevented^9,10^. Currently-used treatments fail to control the convulsions associated with glutamate excitotoxicity following the initial step of excessive cholinergic activation^11^. Notably, antagonists of *N*-methyl-d-aspartate (NMDA)^12^ or AMPA (α-amino-3-hydroxy-5-methyl-4-isoxazolepropionic acid) receptors^13^, two subtypes of central glutamate receptors, afforded additional protection ^14^. In this respect, it is important to note that non-human primates poisoned with 3–5 LD_50_ of soman and treated with a mixture of an oxime, atropine, and benactyzine (TAB), showed remarkable recovery^15,16^. Benactyzine is a compound with mixed anti-cholinergic and anti-glutamatergic properties, approved for emergency use in Israel in OP poisoning^17,18^.

Numerous studies have shown that exposure to different OPs at different doses produces a neuronal loss in various CNS structures, particularly in the entorhinal and piriform cortex, amygdala, and the CA1 and CA3 subfields of the hippocampus^19,20^. It was recently suggested that the degree of brain damage and ensuing cognitive deficits depend on the severity of convulsions^14,21^.

Spatial learning and memory impairments are two major detrimental consequences of OP poisoning, with ensuing neuronal degeneration in CNS structures (e.g., hippocampus). Indeed, cognitive weakness, such as impaired learning and memory resulting from exposure to OPs, was reported in humans and animals^22,23^.

Raveh et al.^24,25^, demonstrated that caramiphen, a drug with a mixed anticholinergic and antiglutamatergic profile, affords better protection when compared to that provided by scopolamine, a pure anticholinergic agent when given in a pretreatment paradigm, and that TAB and caramiphen given to soman-exposed animals offered cognitive protection as reflected with the Morris water maze test^26^. These results show that drugs with a pharmacological profile consisting of anticholinergic and antiglutamatergic properties such as caramiphen and benactyzine have an advantage as post-exposure therapies against poisoning by OP agents^27^.

The lack of a side-effect-free anticonvulsant prompts current research efforts aimed at developing better MCMs for OP-induced seizures. Any such drug should be devoid of behavioral side effects as possible, to diminish incapacitation of the casualty and since the drug may be taken inadvertently. Therefore, it is critical to identify drugs that not only counteract the effects of nerve agents but also produce minimal behavioral effects when administered alone.

Gama-Amino-Butyric-Acid (GABA) is the major inhibitory neurotransmitter in the brain and was found to be involved in the reduction of convulsions^28^. It is known that benzodiazepines that bind allosterically to GABA-chloride channels, display anticonvulsant activity and are commonly administered to patients affected by OP agents. Moreover, the benzodiazepine drug midazolam was shown to be superior over other compounds in terminating OP-induced seizures, with some neuroprotective qualities. However, GABA itself does not readily cross the blood–brain-barrier (BBB) and is therefore ineffective when systemically administered.

Therefore, a concept of a prodrug of an anticholinergic compound acting in the CNS together with GABA activity may offer a novel therapeutic approach, influencing the main receptorial systems involved in seizure initiation and propagation induced by OP poisoning. Moreover, it is anticipated that the inhibitory action of GABA receptors would attenuate some of the central anticholinergic adverse effects (e.g., disorientation, confusion, hallucinations), thus increasing current drugs' efficacy and safety.

Based on our previously described prodrugs development such as the perphenazine-GABA ester for the treatment of schizophrenia^29,30^ and the nortriptyline-GABA amide for the treatment of neuropathic pain^31^, we sought to synthesize a benactyzine-GABA mutual prodrug (labeled "gabactyzine"). It is expected to be able to cross the BBB due to the lipophilic character of the benactyzine, acting as the “carrier” and transporting GABA into the brain. Once the molecule crosses the BBB it should undergo hydrolysis releasing concomitantly benactyzine and GABA, which are expected to act synergistically.

## Methods

### Chemistry (general)

^1^H and ^13^C NMR spectra were obtained on Bruker Avance-200, Avance-DPX-300, Avance-400, Avance-DMX-600, and Avance-III-700 spectrometers. Chemical shifts are expressed in ppm downfield from Me_4_Si (TMS) used as internal standard. The values are given in δ scale. It should be noted that the compounds that have carbamates, frequently are seen in the ^1^H NMR and ^13^C NMR spectra as mixtures of pairs of rotamers. Whereby the signals for the protons of the CH_2_’s, and the corresponding carbons appear in close proximity as pairs of signals. In these cases. the chemical shifts of the rotameric H’s and C’s are indicated while being linked by + signs. For some compounds, the NMR data assignment was aided by several 2-dimensional spectra including COSY, HMQC, and HMBC analyses. High-resolution mass spectra (HRMS) were obtained on a 6545 QTOF instrument (Agilent). The progress of the reactions was monitored by TLC on silica gel (Merck, Art. 5554). All the flash chromatographic procedures were carried out on silica gel (Merck, Art. 9385). The nomenclature of the compounds was assigned according to ChemDraw Ultra version 12.0.3.1216 (Cambridge Soft).

#### General procedure 1: synthesis of esters^32^

A mixture of an alkyl halide (1 mmol), an acid (1 mmol) (*N*-Boc-GABA or 2-(4-(*tert*-butoxycarbonylamino)butanoyloxy)-2,2-diphenylacetic acid, **7**) and DBU (1 mmol) in ~ 2 mL of CH_3_CN (dried over molecular sieves) was stirred at room temperature overnight. The mixture was then quenched with distilled water. A white solid that precipitated was washed with ether, and the organic layer was washed with saturated KHSO_4_. The organic layer was dried over Na_2_SO_4_, filtered, and evaporated. The residue was purified by silica gel chromatography, or by preparative HPLC.

#### General procedure 2: N-Boc removal

To a solution of a Boc-protected amino compound in dry ether was added HCl(g). The solution was stirred for 1–2 h and the solvent was evaporated to give the hydrochloride salt of the free amino compound.

#### α-Bromo-α,α-diphenylacetic acid (16)

A suspension of benzilic acid^33^ in HBr/AcOH was kept at 0 °C overnight. The solvent was evaporated, and the residue was dried under a high vacuum. ^1^H NMR (300 MHz, acetone-*d*_*6*_): δ 7.34–7.40 (m, 6H, ar), 7.48–7.52 (m, 4H, ar). ^13^C NMR (50 MHz, acetone-*d*_*6*_): δ 71.3 (*C*-4°), 128.7, 129.0, 130.0 (*C*H ar), 142.1 (*C*-4°), 171.3 (*C*-4°).

#### 12-(2-(Diethylamino)ethoxy)-2-oxo-1,1-diphenylethyl 4-aminobutanoate dihydrochloride (gabactyzine) (1)

Compound **1** was obtained from **6** as an oil in quantitative yield using procedure 2. ^1^H NMR (300 MHz, MeOD-*d*_*4*_): δ 1.16 (t, *J* = 7.3 Hz, 6H), 1.95 (quint, *J* = 7.7 Hz, 2H), 2.68 (t, *J* = 7.3 Hz, 2H), 2.90 (t, *J* = 7.3 Hz, 2H), 3.03 (q, *J* = 7.6 Hz, 4H), 3.43 (m, 2H), 4.54 (m, 2H), 7.35 (m, 6H, ar), 7.52 (m, 4H, ar). ^13^C NMR (75 MHz, MeOD-*d*_*4*_): δ 9.3 (Me), 23.7, 32.1, 39.8, 49.3, 51.2, 61.5 (six *C*H_2_), 85.6 (C-4°), 128.6, 129.59, (CH ar), 140.55 (C-4°), 170.1 (C-4°), 172.6 (C-4°)*.* HRMS calcd for C_24_H_33_N_2_O_4_^+^ [MH^+^] 413.2435, found 413.2437.

#### 2-(2-(2-(Diethylamino)ethoxy)-2-oxo-1,1-diphenylethoxy)-2-oxo-1,1-diphenylethyl 4-Aminobutanoate dihydrochloride (22)

Compound **22** was obtained from **20** in quantitative yield using procedure 2. ^1^H NMR (700 MHz, MeOD-*d*_*4*_): δ 1.09 (t, *J* = 7.3 Hz, 6H), 1.95 (quint, *J* = 7.7 Hz, 2H), 2.70 (t, *J* = 7.3 Hz, 2H), 2.79 (q, *J* = 7.3 Hz, 4H), 2.91 (br t, *J* = 8.0 Hz, 2H), 3.33 (m, 2H), 4.45 (m, 2H), 7.24 (m, 4H, ar), 7.29 (m, 6H, ar), 7.34 (m, 6H, ar), 7.49 (m, 6H, ar). ^13^C NMR (175 MHz, MeOD-*d*_*4*_): δ 9.14 (Me), 23.8, 32.3, 39.7, 39.7, 49.8, 51.0 (six *C*H_2_), 86.7 (C-4°), 87.0 (C-4°),128.5, 129.12, 129.22, 129.25, 129.77 (CH ar), 139.50 (C-4°), 140.3 (C-4°), 168.1 (C-4°), 169.6 (C-4°), 172.23 (C-4°)*.*

#### 2-(2-(2-(Diethylamino)ethoxy)-2-oxo-1,1-diphenylethoxy)-2-oxo-1,1-diphenylethyl 4-(tert-butoxycarbonylamino)butanoate (20)

Compound **20** was obtained from **18** as an oil using procedure 1. ^1^H NMR (700 MHz, acetone-*d*_*6*_): δ 1.12 (t, *J* = 7.3 Hz, 6H), 1.41 (s, 9H), 1.84 (quint, *J* = 7.7 Hz, 2H), 2.68 (t, *J* = 7.8 Hz, 2H), 3.02 (br q, 4H), 3.11 (t, = 7.0 Hz, 2H), 3.41 (br t, 2H), 4.55 (m, 2H), 6.15 (br t, NH), 7.23–7.29 (m, 7H, ar), 7.35–7.36 (m, 9H, ar), 7.55–7.57 (m, 4H, ar). ^13^C NMR (175 MHz, acetone-*d*_*6*_): δ 8.8 (Me), 26.0 (*C*H_2_), 28.6 (*Boc Me’s*), 32.4, 40.17, 47.7, 50.0, 60.8 (five *C*H_2_), 78.5 (*C*-4°), 85.07 (*C*-4°), 85.93 (*C*-4°), 127.87, 128.44, 128.84, 128.89, 129.09 (*C*H ar), 139.36 (*C*-4°), 139.36 (*C*-4°), 156.69 (*C*O carbamate), 167.55 (*C*-4°), 168.9 (*C*-4°), 172.15 (*C*-4°)*.*

#### 2-(2-(Diethylamino)ethoxy)-2-oxo-1,1-diphenylethyl 4-(tert-butoxycarbonylamino)butanoate (6)

Compound **6** was obtained from **7** as an oil in ~ 45% using procedure 1. ^1^H NMR (700 MHz, acetone-*d*_*6*_): δ 0.94 (t, *J* = 7.2 Hz, 6H), 1.41 (s, 9H), 1.85 (quint, *J* = 7.2 Hz, 2H), 2.36–2.64 (m, 8H), 3.14 (q, *J* = 6.4 Hz, 2H), 4.17 (t, = 6.1 Hz, 2H), 6.05 (br s, 1H), 7.28–7.38 (m, 6H, ar), 7.57–7.61 (m, 4H, ar). ^13^C NMR (75 MHz, acetone-*d*_*6*_) δ 12.5 (Me), 26.1 (*C*H_2_), 28.6 (*Boc Me’s*), 32.4, 40.4, 48.0, 51.8, 64.8 (five *C*H_2_), 78.5 (*C*-4°), 84.7 (*C*-4°), 128.25, 128.58, (*C*H ar), 141.4 (*C*-4°), 156.7 (*C*O carbamate), 169.8 (*C*-4°), 171.9 (*C*-4°).

#### 3-Ethyl-7,10,13-trioxo-8,8,11,11,14,14-hexaphenyl-6,9,12-trioxa-3-azatetradecan-14-yl 4-(tert-butoxycarbonylamino)butanoate (21)

Compound **21** was obtained in trace amounts from **19** as a white solid, using procedure 1. ^1^H NMR (700 MHz, acetone-*d*_*6*_): δ 1.06 (t, *J* = 7.7 Hz, 6H), 1.41 (s, 9H), 1.86 (quint, *J* = 7.0 Hz, 2H), 2.63 (t, *J* = 7.7 Hz, 2H), 2.85 (br s, 4H), 3.12 (t = 7.0 Hz, 2H), 3.20 (br t, 2H), 4.44 (m, 2H), 6.12 (br t, NH), 7.21–7.24 (m, 8H, ar), 7.26–7.30 (m, 14H, ar), 7.43–7.44 (m, 8H, ar). ^13^C NMR (175 MHz, acetone-*d*_*6*_): δ 8.9 (Me), 26.2 (*C*H_2_), 28.6 (*Boc Me’s*), 32.6 (CH_2_), 40.36 + 40.24 (br s, CH_2_ two rotamers), 47.6, (CH_2_), 49.7 (CH_2_), 60.7 (*C*H_2_), 78.5 (*C*-4°), 85.5 (*C*-4°), 86.4 (*C*-4°), 87.1 (*C*-4°), 128.54, 128.59, 128.66, 128.73, 128.86, 129.02, 129.09, 129.37 (*C*H ar), 139.16 (*C*-4°), 139.38 (*C*-4°), 140.38 (*C*-4°), 156.69 + 159.73 (*C*O carbamate two rotamers), 166.47 (*C*-4°), 167.40 (*C*-4°), 168.98 (*C*-4°), 171.32 (*C*-4°)*.*

#### 2-(4-(tert-Butoxycarbonylamino)butanoyloxy)-2,2-diphenylacetic acid (7)

Compound **7** was obtained from **16** as an oil using procedure 1 and was used further without additional purification. ^1^H NMR (200 MHz, acetone-*d*_*6*_): δ 1.40 (s, 9H), 1.84 (quint, *J* = 6.9 Hz, 2H), 2.55 (t, *J* = 6.6 Hz, 2H), 3.12 (q, *J* = 6.6 Hz, 2H), 6.05 (br s, 1H), 7.29–7.35 (m, 6H, ar), 7.62–7.66 (m, 4H, ar). ^13^C NMR (75 MHz, acetone-*d*_*6*_): δ 26.0 (*C*H_2_), 28.6 (*Boc Me’s*), 32.3 (*C*H_2_), 40.3 (*C*H_2_), 78.6 (*C*-4°), 84.4 (*C*-4°), 128.23, 128.60, (*C*H ar), 141.6 (*C*-4°), 156.8 (*C*O carbamate), 170.7 (*C*-4°), 171.8 (*C*-4°).

#### Benzhydryl 4-(tert-butoxycarbonylamino)butanoate (17)

Compound **17** was obtained from **16** as an oil in 38% using procedure 1 in refluxing benzene. ^1^H NMR (700 MHz, acetone-*d*_*6*_): δ 1.39 (s, 9H), 1.81 (quint, *J* = 7.7 Hz, 2H), 2.51 (t, *J* = 7.7 Hz, 2H), 3.15 (q, *J* = 6.3 Hz, 2H), 6.85 (br s, 1H, NH), 6.85 (s, 1H, C*H*), 7.28 (m, 2H, ar), 7.35 (m, 2H, ar), 7.41 (m, 2H, ar). ^13^C NMR (75 MHz, acetone-*d*_*6*_): δ 26.1 (*C*H_2_), 28.6 (Boc *Me’s*), 32.0 (*C*H_2_), 40.19 + 40.32 (*C*H_2_ two rotamers), 77.4 (*C*H), 78.4 (*C*-4°), 127.6, 128.5, 129.3 (*C*H ar), 141.8 (*C*-4°), 156.7 (*C*O carbamate), 172.5 (*C*-4°).

#### 2-(2-(Diethylamino)ethoxy)-2-oxo-1-phenylethyl 4-(tert-butoxycarbonylamino)butanoate (13)

Compound **13** was obtained from **12** as an oil in 79% using procedure 1. ^1^H NMR (200 MHz, acetone-*d*_*6*_): δ 1.02 (t, *J* = 7.2 Hz, 6H), 1.51 (s, 9H), 1.95 (quint, *J* = 7.2 Hz, 2H), 2.55 (q, *J* = 7.2 Hz, 4H), 2.72 (t, *J* = 6.0 Hz, 2H), 3.25 (q, *J* = 6.4 Hz, 2H), 4.26 (m, 2H), 6.20 (br s, 1H), 7.54–7.53 (m, 3H, ar), 7.63–7.65 (m, 2H, ar). ^13^C NMR (75 MHz, acetone-*d*_*6*_): δ 12.5 (Me), 26.1 (*C*H_2_), 28.6 (*Boc Me’s*), 31.7, 40.7, 48.0, 51.9, 64.6 (five *C*H_2_), 75.3 (*C*H), 78.5 (*C*-4°), 128.6, 129.4, 129.8 (*C*H ar), 135.2 (*C*-4°), 156.6 (*C*O carbamate), 169.4 (*C*-4°), 173.0 (*C*-4°).

#### N-Bromo-N,N-diethyl-2,2-dimethyl-4,9,12-trioxo-11-phenyl-3,10,13-trioxa-5-azapentadecan-15-aminium (15)

*Method 1* To a solution of** 13** (1 eq) in dry EtOAc, CuBr_2_ (1 eq) was added. The mixture was refluxed for 1 h, and when no starting material was detected by TLC, the mixture was filtered through a short silica column. The silica was washed EtOAc/MeOH, and the combined eluent was evaporated to give compound **15** as an oil in quantitative yield. *Method 2* To a solution of** 13** (1 eq) in CCl_4_ was added *N*-bromosuccinimide (NBS) (2 eq) and azobisisobutyronitrile (AIBN) (0.025 eq). The solvent was evaporated after 1.3 h when starting material was not detected by TLC. ^1^H NMR (200 MHz, acetone-*d*_*6*_): δ 1.38 (m, 15H, *Boc Me’s*, two C*H*_3_), 1.82 (quint, *J* = 7.0 Hz, 2H), 2.51 (t, *J* = 7.4 Hz, 2H), 3.14 (q, *J* = 6.4 Hz, 2H), 3.46 (q, *J* = 7.2 Hz, 4H), 3.78 (br t, *J* = 6.9 Hz, 2H), 4.66 (m, 2H), 6.05 (s, 1H) 6.20 (br s, 1H), 7.54–7.53 (m, 3H, ar), 7.63–7.65 (m, 2H, ar). ^13^C NMR (75 MHz, acetone-*d*_*6*_): δ 8.4 (Me), 25.0 (*C*H_2_), 27.6 (*Boc Me’s*), 30.0, 39.1, 48.6, 50.7, 59.7 (five *C*H_2_), 74.0 (*C*H), 78.0 (*C*-4°), 127.6, 128.7, 129.3 (*C*H ar), 137 (*C*-4°), 156.6 (*C*O carbamate), 169.8 (*C*-4°), 172.3 (*C*-4°).

#### 2-(Diethylamino)ethyl 2-bromo-2-phenylacetate (12)

A solution of** 11** (1 eq) and diethylaminoethanol in CH_2_Cl_2_ was stirred at room temperature overnight and was then washed with 5% aq. NaHCO_3_, dried over Na_2_SO_4_, filtered and evaporated to give compound **12** as a yellow oil in 72%. ^1^H NMR (300 MHz, acetone-*d*_*6*_): δ 0.93 (t, *J* = 6.9 Hz, 6H), 2.45 (q, *J* = 6.9 Hz, 4H), 2.64 (t, *J* = 6.0 Hz, 2H), 4.13 (t, *J* = 6.9 Hz, 2H), 5.67 (s, 1H), 7.39–7.41 (m, 3H, ar), 7.56–7.59 (m, 2H, ar). ^13^C NMR (75 MHz, acetone-*d*_*6*_): δ 12.5 (Me), 47.9 (*C*H_2_), 51.7(*C*H_2_), 59.7 (CH), 65.2 (*C*H_2_), 75.3 (*C*H), 128.8, 129.4, 129.8 (*C*H ar), 137.2 (*C*-4°), 168.6 (*C*-4°).

### Biology (general)

#### Animals

The study was approved by and performed in accordance with the guidelines of The Animal Care and Use Committee of Sheba Medical Center, which is approved by the Israeli authorities for animal experimentation. The reporting in the manuscript follows the recommendations in the ARRIVE guidelines.

Adult male albino Sprague–Dawley rats, weighing 300–320 g (Harlan-biotech, Jerusalem, Israel), were used in all studies, following two–three days of acclimatization in the animal facility. The animals were housed under standard laboratory conditions in plastic cages, four per cage at a temperature of 21 ± 2 °C and 50 ± 10% humidity, in controlled animal quarters, and maintained on a 12 h light–dark cycles (light on at 6.00 a.m.). Food and water were available ad labium.

#### Drugs

Paraoxon, atropine sulfate, obidoxime, and midazolam were purchased from Sigma (Israel). Paraoxon was diluted in a vehicle containing 40% propylene glycol, 10% EtOH, 1.5% benzyl alcohol and 48.5% distilled H_2_O. All other drugs were diluted in normal saline. The novel compound gabactyzine was synthesized and supplied by the Medicinal Chemistry laboratory of Bar-Ilan University. Anesthesia applied before each imaging session, was administered by an intramuscular (IM) injection consisting of 450 µL of Ketamine (22.5 mg/mL) and 150 µL xylazine (0.3%).

#### Study no 1: a preliminary safety study

A preliminary observational study was performed in naïve rats to evaluate the gross toxic effects of the compound during a relatively short period, serving as a go-no go approach. We used escalating doses of gabactyzine ranging from 1 to 5.2 mg/kg, injected IM, and continuously observed the treated animals for a period of 6 h, regarded as sufficient for this stage of development of an antidotal therapy, allowing to assess any potential acute adverse effects of the drug^34^.

#### Study no 2: a preliminary efficacy study

##### Experimental outline

This study focused on evaluating the efficacy of gabactyzine in a rat model of paraoxon poisoning using two treatment schedules. Adult (8-week-old) male albino Sprague–Dawley rats weighing 300–320 g (Harlan-biotech, Jerusalem, Israel) were housed under standard laboratory conditions in plastic cages, four per cage in a controlled environment with constant temperature of 21_2C_ and a 12 h light/dark cycle. Food (Teklad certified global 18% protein) and water were available ad libitum. Care and maintenance were in accordance with the principles described in the Guide for Care and Use of Laboratory Animals (NIH publication no. 85–23, 1985). The experimental protocol was examined and approved by the Institutional Committee for Animal Experimentation as required by local law. Paraoxon, atropine sulfate, and obidoxime were purchased from Sigma–Aldrich, Jerusalem, Israel. Paraoxon was diluted in a vehicle containing 40% propylene glycol. All other drugs were diluted in normal saline. 24 animals were exposed to 1.4 LD_50_ of paraoxon (450 μg/kg, IM, 0.5 mL/kg). One min after exposure, all rats were treated with atropine (3 mg/kg, IM, 0.5 mL/kg) and obidoxime (20 mg/kg, IM, 0.5 mL/kg). One min or 30 min after the clinical appearance of general tonic–clonic convulsions (2–3 min post poisoning, n = 8 in each group), gabactyzine (5.2 mg/kg) was administered IM. The use of 1- and 30-min time points were previously established in the rat model of paraoxon poisoning as allowing to test the efficacy of anticonvulsant drugs^35,36^, representing either immediate use of an autoinjector, or if delayed, representing treatment given by a medic in the field. The paraoxon dose was chosen based on previous studies, showing it to be toxic enough to induce seizures, and with the chosen atropine and oxime doses will allow survival but will not prevent the brain damage, thus allowing to test the efficacy of various centrally-acting drugs in alleviating the brain injury^35,36^. The 5.2 mg/kg gabactyzine dose was chosen as this was the highest dose tested showing no adverse effects. The animals were closely observed for post-exposure clinical signs every 15 min for a period of 4 h, and then once a day for a week. Observation included signs of cholinergic toxicity and of general distress (eye secretion, diarrhea, fasciculations, tremor, convulsions, dyspnea, decreased motor activity, ataxia, Straub tail, piloerection, chewing, etc.). The clinical signs were processed into a scoring system of 0–4: 0 = no reaction, 1 = mild reaction (animal exhibited Straub tail, peri-orbital staining or peri-nasal staining, piloerection, chewing and/or ataxia), 2 = moderate reaction (animal exhibited decreased motor activity, hunched posture, dyspnea, eye secretions and/or ptosis in addition to the above-mentioned clinical signs), 3 = severe reaction (animal exhibited abdominal position, fasciculations, tremors, jerks, convulsions and/or dehydration in addition to the above mentioned clinical signs), 4 = mortality. The rats were weighed before exposure and daily thereafter.

#### Study no 3: efficacy study

##### Experimental outline

Sixty adult (8-week-old) male albino Sprague–Dawley rats weighing 300–320 g (Harlan-biotech, Jerusalem, Israel) were handled as in the second study depicted above. All animals were administered a dose of 1.4 LD_50_ of Paraoxon (450 µg/kg, IM, 0.5 mL/kg). One min after poisoning, all rats were treated with Atropine (3 mg/kg, IM, 0.5 mL/kg) and Obidoxime (20 mg/kg, IM, 0.5 mL/kg). Either 1 or 30 min after clinical generalized seizures were evident, one of three anticonvulsant drugs—midazolam (1 mg/kg), benactyzine (1 mg/kg) or gabactyzine (1 mg/kg)—was administered IM. The clinical state was assessed and scored every 15 min until 1 h after poisoning, and then once per day. The rats were divided into 5 treatment groups:

- Group C served as control (n = 12): No additional treatment
- Group G1 was treated with gabactyzine 1 min post poisoning (n = 8)
- Group B1 was treated with benactyzine 1 min post poisoning (n = 9)
- Group G30 was treated with gabactyzine 30 min post poisoning (n = 18)
- Group M30 was treated with midazolam 30 min post poisoning (n = 13)
- Group B30 was treated with benactyzine 30 min post poisoning (n = 9)

The animals were scanned by MRI 1-, 8-, and 15-days post-exposure. Anesthesia, applied prior to each imaging session, was administered by an intra-muscular (i.m.) injection consisting of ketamine (450 mL, 22.5 mg/mL) and Xylasine (150 mL, 0.3%). The rats were weighed before treatment and following each MRI scan. At the end of the follow-up, the rats were sacrificed.

MR imaging and analysis methods of T2-weighted MRI, diffusion weight MRI (DWMRI), and apparent diffusion coefficient maps followed the methods described in Rosman et al.^35^ and in Gudbjartsson et al.^37^. In short, scanning was performed using a General Electric 3.0 T MRI machine (GE Medical Systems, Waukesha, WI, USA) with the HD12 operating system, gradients intensity of up to 4.3 Gauss/cm, the line scan diffusion-weighted imaging acquisition software package (LSDI)^37^, and a clinical phased array knee coil.

T2-weighted fast spin echo (FSE) MR images were acquired with: 256 × 128 matrix, 12 × 9 cm^2^ field of view (FOV), repetition time (TR) of 3000 ms, echo time (TE) of 90 ms and 2 mm slices covering the full rat brain, thus containing the 3D information. LSDI DWMR images were acquired with: 256 × 128 matrix, 12 × 9 cm^2^ FOV, TR = 5440 ms, TE = 142 ms and 2 mm slices. Data was acquired at b = 0 and 1000 s/mm^2^. Volumes of the temporal tissue toxicity were calculated by plotting regions of interest (ROIs) over the entire enhancing regions in each DWMR slice, adding the number of pixels over the slices and multiplying by the volume of each pixel. Ventricular volume representing hydrocephalus was calculated by plotting ROIs over the frontal ventricles in each T2-weighted MR slice, adding the number of pixels over the slices and multiplying by the volume of each pixel.

Same scores used in the previous study were implemented here, ranging from 0 (no reaction) to 4 (mortality). The numbers of animals are shown in parenthesis. The percentage was calculated from surviving animals in each treatment group for that day.

### Statistical analysis

Analysis was performed using the SPSS^®^ computer program (version 17, IBM, Armonk, NY, USA). In the second study, the clinical score was subjected to a two-way analysis of variance (ANOVA) with a repeated measure on one variable (time following paraoxon exposure), with p < 0.05 considered statistically significant (marked with *); a 1-Way ANOVA Dunnett Multiple Comparison Test was used to analyze the change in weight, with p < 0.05 considered statistically significant. Survival in the third study was analyzed using Kaplan–Meier survival curves. Comparison between groups was performed using the unpaired two-tailed t-test, except where the changes in the Apparent Diffusion Coefficient (ADC) as a function of time were studied. In this case, the ADC of each rat was compared at different time points, thus the one-tailed paired t-test was used.

## Results

### Chemistry

**Figure 1:**
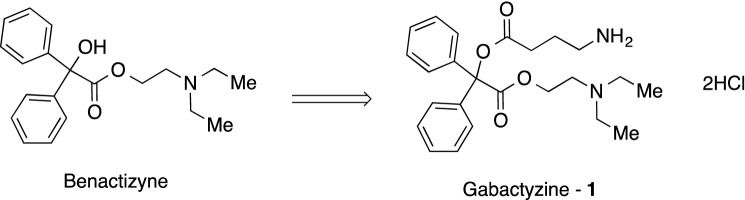
The synthesis phase. The aim of the synthesis phase was to develop Gabactyzine from the combination of benactyzine and Gama-Amino-Butyric-Acid (GABA).

The synthetic goal of this study was the development of a suitable process for the preparation of gabactyzine (Fig. 1).

Although gabactyzine is the GABA ester of benactyzine, all attempts to directly esterify benactyzine with carboxy-activated-*N*-protected GABA derivatives or with GABA itself under acidic conditions, failed. A search of the literature revealed that the only esters of benactyzine reported are the acetate and propionate for which no synthetic procedures are provided^38–42^. The highly hindered character of the 3^ary^ OH group of benactyzine is reflected in a variety of unsuccessfully attempted acylation conditions using i.e., benzotriazol-1-yloxytripyrrolidinophosphonium hexafluorophosphate (Py-Bop), 1-ethyl-3-(3-dimethylaminopropyl)carbodiimide (EDCI), 2,4,6-trichlorobenzoyl chloride (TCBC—Yamaguchi reagent), 1-hydroxy-7-azabenzotriazole (HOAt)/(1-[bis(dimethylamino)methylene]-1H-1,2,3-triazolo[4,5-b]pyridinium 3-oxide hexafluorophosphate (HATU), or GABA + benactyzine in the presence of a catalytic amount of concentrated H_2_SO_4_^43^ with *N*-Boc-GABA, where no evidence of esterification was detected.

Because of our inability to carry out direct esterification of benactyzine, various approaches were undertaken to build intermediates suitable for modification, which would lead to the desired gabactyzine. The synthesis originally expected to be a straightforward one turned out to be quite challenging.

**Figure 2:**
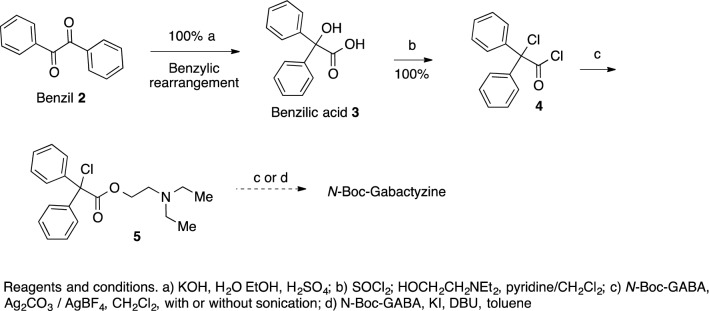
The synthesis phase. Attempted synthesis of intermediate 6.

Benzylic acid was converted to the corresponding α-chloroacyl chloride **4**, which was esterified with diethylaminoethanol to give the corresponding α-chloro ester **5**^44^. Several unsuccessful attempts were then made to replace the chloride with *N*-Boc-GABA (Fig. 2).

**Figure 3:**
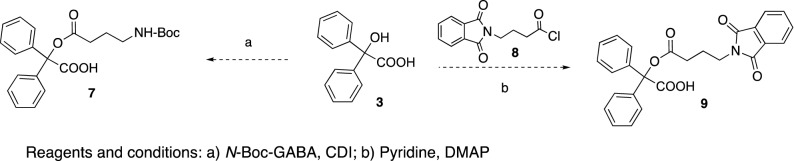
The synthesis phase. Attempted acylations of benzilic acid.

Switching the order of the reactions, by initial esterification of benzylic acid **3** with *N*-Boc-GABA to give the GABA-benzylic ester **7,** or with the phthalimido derivative **8** to give the ester **9,** failed again, probably because of the hindered steric character of the OH group in benzylic acid (Fig. 3).

**Figure 4:**
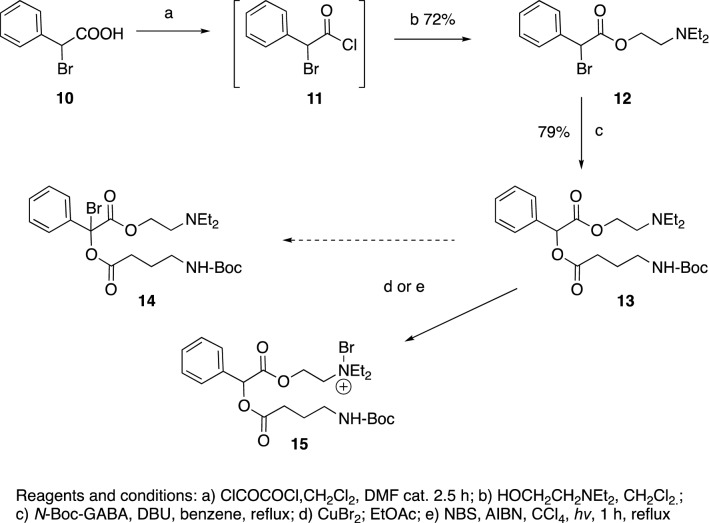
The synthesis phase. An alternative approach to achieve acylations of benzilic acid.

To facilitate the acylation step, an alternative approach was undertaken whereby the α-bromophenylacetic acid **10** was converted into the corresponding acyl chloride **11**^45^, which subsequently was treated with diethylaminoethanol gave the ester **12**^46^. The bromide in compound **12** was then replaced with *N*-Boc-GABA in the presence of the dehydrohalogenating base DBU, to give **13**. Next, we planned to conduct a photolytic bromination at the benzylic position using CuBr_2_ or NBS/hν expecting to obtain **14**, which would then be followed by a Suzuki reaction with phenylboronic acid. However, the only product isolated was **15**, formed by bromination of the nitrogen instead of the benzylic carbon (Fig. 4).

**Figure 5:**
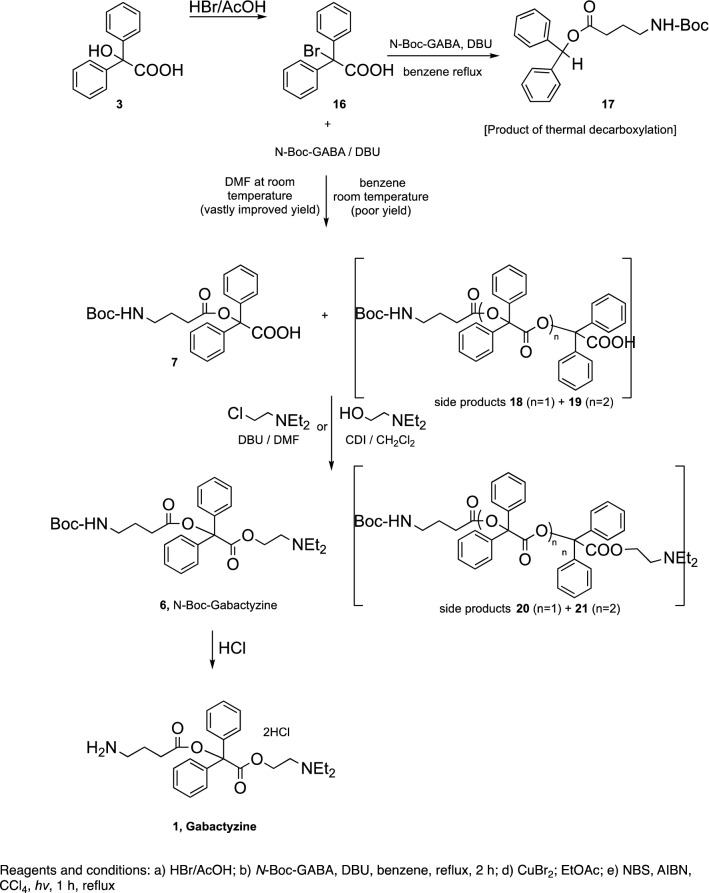
The synthesis phase. The final synthesis of gabactyzine.

An alternative strategy was then developed to avoid the poor nucleophilic character of the 3^ary^ OH of benactyzine, by converting the benzylic position into a highly electrophilic carbocation. This approach led to the successful synthesis of gabactyzine as shown in Fig. 5.

Benzilic acid was converted into the α-bromo derivative **16**^47^ when treated with HBr/acetic acid. The bromide was then replaced with the *N*-Boc-GABA fragment when **16** was treated with 1,8-diazabicyclo[5.4.0]undec-7-ene (DBU) in refluxing benzene^32^. However, since the reaction was carried out at reflux temperature, most of the isolated material was the product of thermal decarboxylation **17.** Whereas in previous attempts, we encountered great difficulty in direct esterification of the 3^ary^ OH group of benactyzine, herein the condensation between the *N*-Boc-GABA/DBU and **16** proceeded readily involving replacement of Br by the *N*-Boc-GABA carboxylate, to give **7**, albeit in low yield. The yield of **7** was improved considerably when the reaction was carried out in DMF or CH_3_CN at room temperature to give **7** together with small amounts of the side products **18** and **19.** It is assumed that **18** and **19** were obtained by an S_N_1 reaction involving the formation of the planar carbocation, which reacted with the *N*-Boc-GABA/DBU. Treatment of the mixture **7**, **18** and **19** with 2-diethylaminoethyl chloride hydrochloride and two additional equivalents of DBU, or with 2-diethylaminoethanol/carbonyldiimidazole (CDI), led to the formation of the *N*-Boc-protected gabactyzine **6**, together with **20** and **21**. Removal of **20** and **21** by silica gel chromatography was unsuccessful and therefore these side products were removed by preparative HPLC. Final deprotection of the Boc group from **6** in the presence of dry HCl(g) gave the desired gabactyzine **1**.

### Biological studies

#### Study number 1: a preliminary study

No changes from baseline activities were noted and no clinical adverse effects were observed. We concluded that the compound does not have observable adverse effects on animals' behavior in the doses used.

#### Study number 2

**Figure 6:**
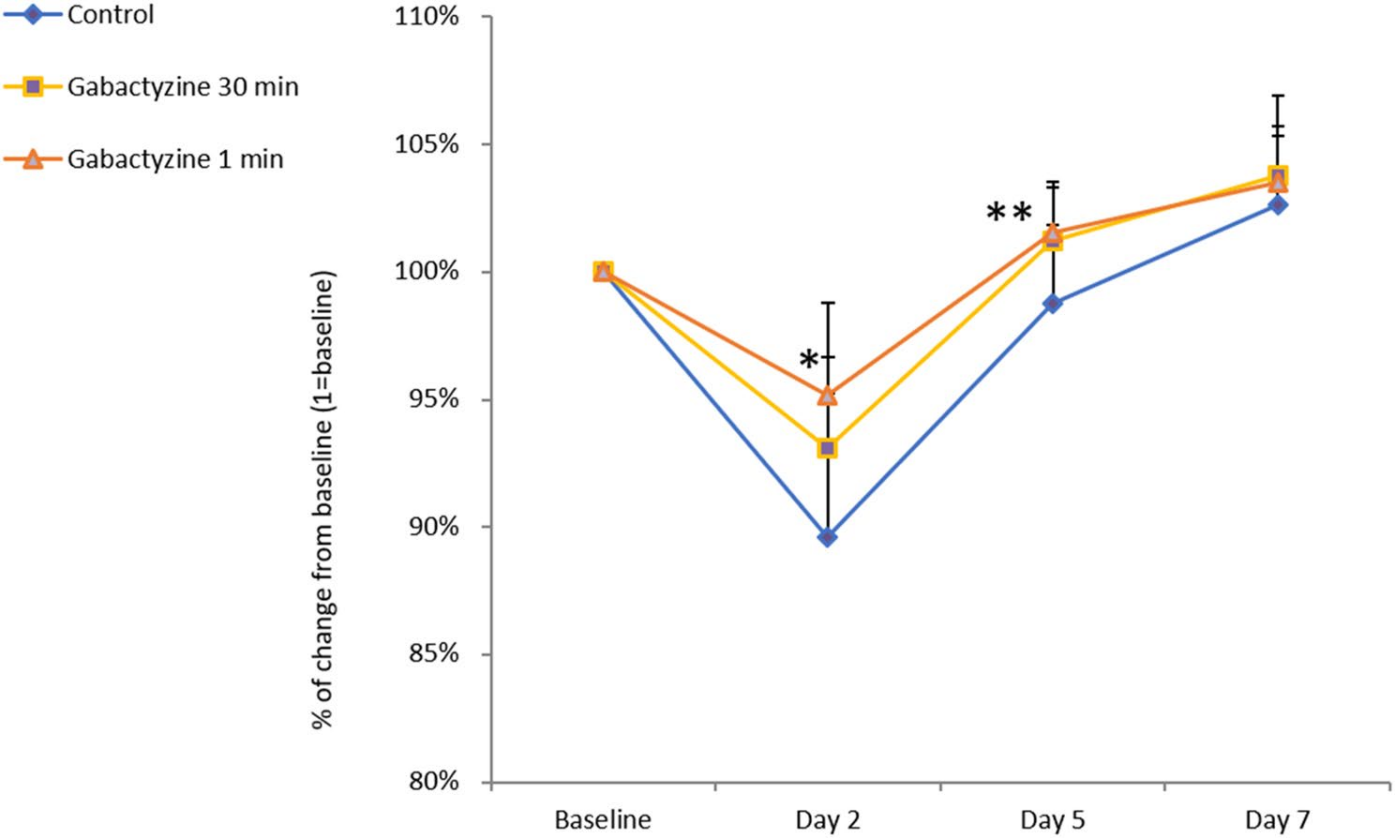
Relative change in weight of animals from baseline to endpoint in study number 2 (7-day follow-up). Comparisons were performed between the control group, gabactyzine 1 min treatment group, and gabactyzine 30 min treatment group (n = 8 in each group) using the 1-Way ANOVA Dunnett Multiple Comparison Test. Significant changes were found between the 1 min treatment group and the control group on day 2 (*p < 0.01) and day 5 (**p < 0.05) following treatment.

After paraoxon injection, all animals convulsed. In both the control group and the gabactyzine—30 min treatment group, one animal was euthanized 48 h after exposure due to extreme weight loss from baseline. The change in the rats' weights as a function of time is shown in Fig. 6. There was a significant difference in weight loss between the groups during the follow-up week. The clinical scores of each treatment group are shown in Table 1. Twenty-four h following exposure there were no signs of toxicity in animals that were administered gabactyzine one min after exposure.

**Table 1:** Change in clinical score between groups in the paraoxon model. Twenty-four rats were exposed to 1.4 LD_50_ of paraoxon (450 μg/kg, IM, 0.5 mL/kg). One min after exposure, all rats were treated with atropine (3 mg/kg, IM, 0.5 mL/kg) and obidoxime (20 mg/kg, IM, 0.5 mL/kg). One min or 30 min after the clinical appearance of general tonic–clonic convulsions (2–3 min post poisoning, n = 8 in each group), gabactyzine (5.2 mg/kg) was administered IM. The scoring system was defined as no reaction, mild reaction (animal exhibited Straub tail, peri-orbital staining or peri-nasal staining, piloerection, chewing and/or ataxia), moderate reaction (animal exhibited decreased motor activity, hunched posture, dyspnea, eye secretions and/or ptosis in addition to the above-mentioned clinical signs), severe reaction (animal exhibited abdominal position, fasciculations, tremors, jerks, convulsions and/or dehydration in addition to the above mentioned clinical signs), and mortality. The clinical score was subjected to a two-way analysis of variance (ANOVA) with a repeated measure on one variable (time following paraoxon exposure), with p < 0.05 considered statistically significant (marked with *).

	Clinical score	Day 0	Day 1	Day 2	Day 3
Control	Mean score ± SD	2.4 ± 0.5	2.4 ± 0.7	0.8 ± 1.5	0.3 ± 0.8
	No reaction	–	–	75% (6)	86% (6)
	Mild	–	13% (1)	–	–
	Moderate	63% (5)	38% (3)	13% (1)	–
	Severe	38% (3)	50% (4)	–	–
	Mortality	–	–	13% (1)	14% (1)
Gabactyzine 30 min	Mean score ± SD	2.3 ± 0.5	1.4 ± 1.2	0.5 ± 1.4	0*
	No reaction	–	38% (3)	87% (7)	100% (7)
	Mild	–	–	–	–
	Moderate	75% (6)	38% (3)	–	–
	Severe	25% (2)	25% (2)	–	–
	Mortality	–	–	13% (1)	–
Gabactyzine 1 min	Mean score ± SD	2 ± 0.8	0*	0*	0*
	No reaction	–	100% (8)	100% (8)	100% (8)
	Mild	25% (2)	–	–	–
	Moderate	50% (4)	–	–	–
	Severe	25% (2)	–	–	–
	Mortality	–	–	–	–

#### Study number 3: survival

**Figure 7:**
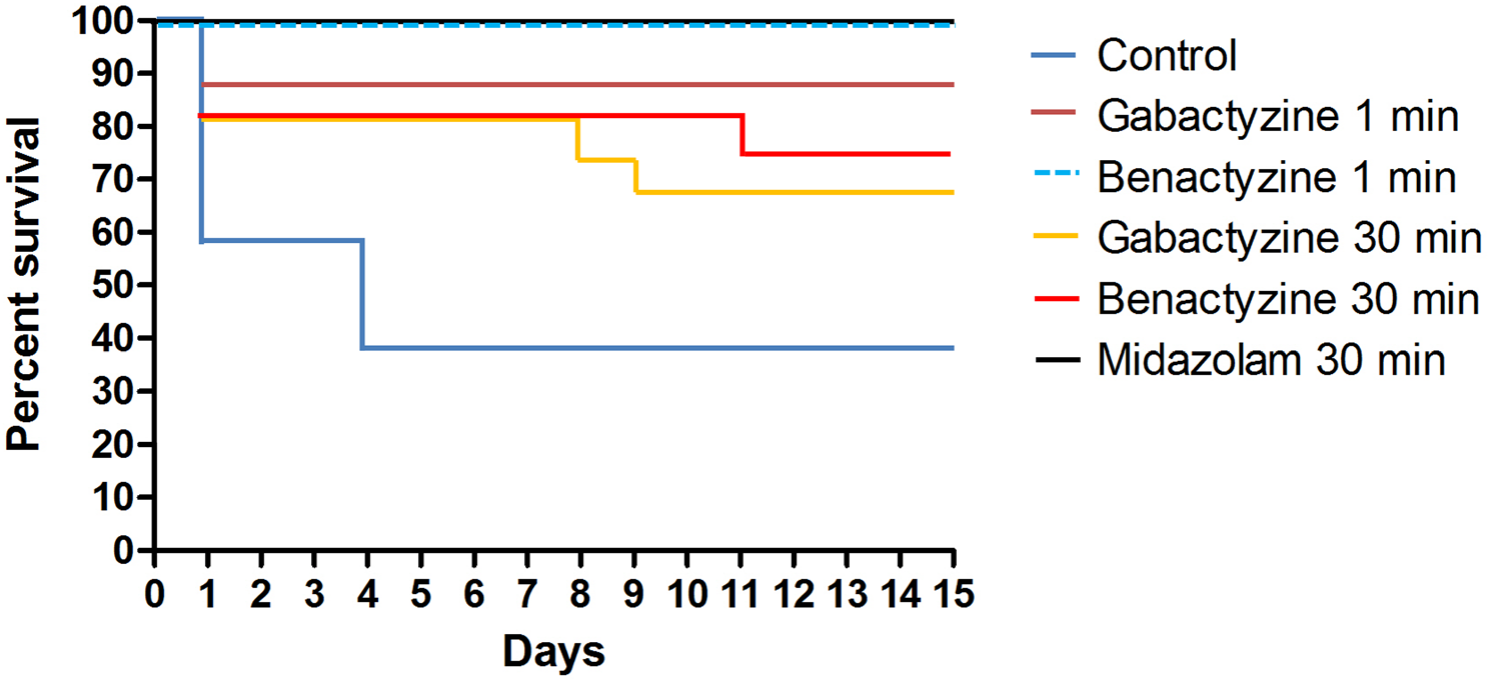
Kaplan–Meier curves of the different treatment groups in study number 3. The survival was significantly higher in benactyzine 1 min (p = 0.0005), midazolam 30 min (p < 0.0001), gabactyzine 1 min (p = 0.0041) and gabactyzine 30 min (p = 0.0041) than in the control group (Wilcoxon test). Between treated groups, only gabactyzine 30 min showed lower survival rates compared to benactyzine 1 min (p = 0.05) and midazolam 30 min (p = 0.02). The curves for groups benactyzine 1 min and midazolam 30 min overlap.

Kaplan–Meier survival curves of the different treatment groups are shown in Fig. 7. The survival was higher in the B1 (p = 0.0005), M30 (p < 0.0001), G1 (p = 0.0041) and G30 (p = 0.0041) groups compared to control (C, Wilcoxon test). Between the treatment groups, only G30 showed lower survival rates compared to B1 (p = 0.05) and M30 (p = 0.02).

#### Tissue toxicity depicted by MRI

**Figure 8:**
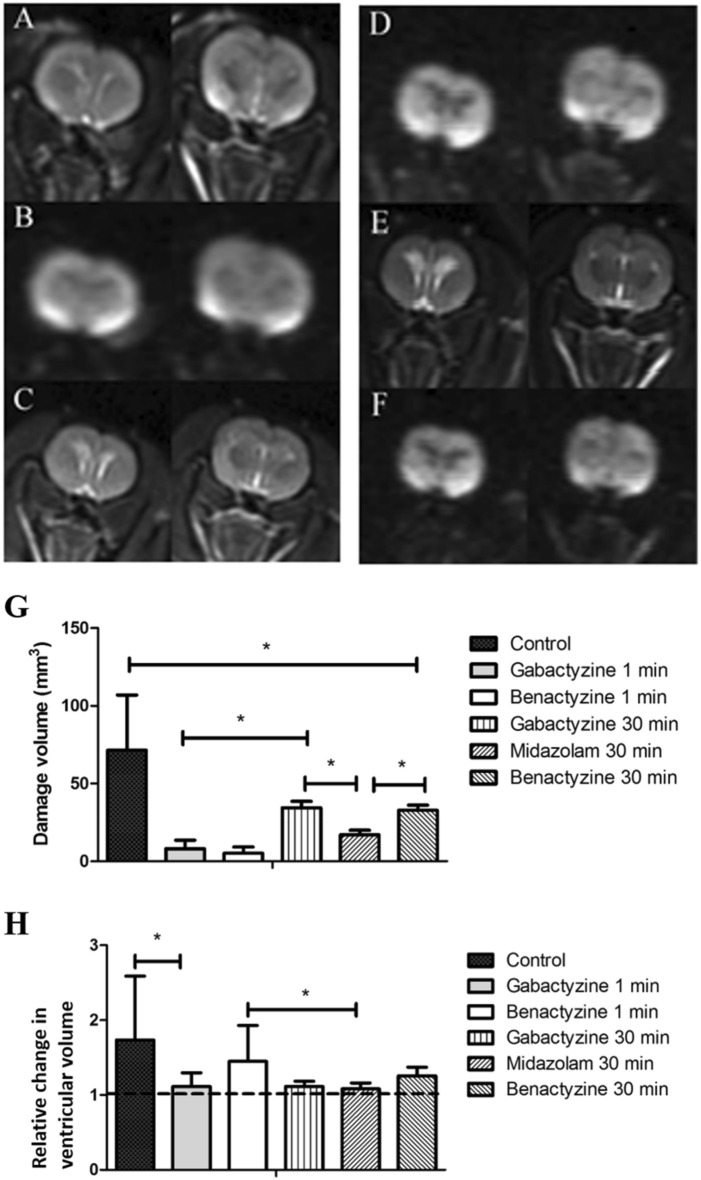
An example of two adjacent axial MR slices of a rat treated with gabactyzine 30 min after poisoning. 1 (**A**,**B**), 8 (**C**,**D**) and 15 (**E**,**F**) days after poisoning (**A**,**C**,**E**: T2-weighted MRI, **B**,**D**,**F**: diffusion-weighted MRI). Significant temporal tissue toxicity (bright signal) is depicted on diffusion-weighted MRI (**B**) and T2-weighted MRI (**A**) 1 day after poisoning. MRI shows moderate ventricular enlargement one day post-treatment and significant ventricular enlargement after 15 days (**E**). (**G**) Average volumes of the bright temporal enhancement on diffusion-weighted MRI for the different treatment groups 24 h post poisoning. The control group showed significantly higher damage volume than all treated groups (p < 0.007). (**H**) Change (ratio) in ventricular volume between day 15 and baseline, for the different treatment groups. There was a significant difference in relative change of ventricular volume between the control and the treatment groups. The benactyzine-treated group showed a tendency towards increased volume enlargement versus other treatments. Dotted line represents the baseline volume level.

Significant tissue toxicity was depicted by diffusion-weighted and T2-weighted MRI 24 h post-treatment, manifested as bright temporal enhancement with low apparent diffusion coefficients. An example is shown in Fig. 8A (DWMRI) and Fig. 8B (T2-weighted MRI).

Figure 8G shows the average volume of the bright temporal enhancement on diffusion-weighted MRI (DWMRI) for the different treatment groups 24 h post poisoning. The volume of temporal damage was significantly higher in the control (71.4 ± 20.5 mm^3^) than in all treatment groups: G1 (8.1 ± 1.9 mm^3^, p = 0.0005), B1 (5.2 ± 1.4 mm^3^, p = 0.0003), G30 (34.0 ± 3.8 mm^3^, p = 0.007) and M30 (17.0 ± 3.0 mm^3^, p = 0.0002).

#### Ventricular enlargement depicted by T2-weighted MRI

Later toxicity, reflected by ventricular enlargement, was noted from the second follow-up and on. An example is shown in Fig. 8C,D (Day 8 post-exposure, DWMRI and T2-weighted MRI, respectively) and Fig. 8E,F (Day 15 post-exposure, DWMRI and T2-weighted MRI, respectively).

Figure 8H shows the change in ventricular volume as a function of time for the different groups. There was no significant difference in relative change of ventricular volume between the treated groups. On day 15, M30 (1.08 ± 0.08) showed a significantly lower relative change of ventricular volume than control (1.73 ± 0.60, p = 0.03).

#### Weight changes

There was no significant difference between the weights of the different groups before poisoning (average weight: 301 ± 2 g), and there was no significant difference between treatment groups during the different follow-ups.

## Discussion

Shortly after exposure to OPs, and even in transient exposure and despite antidotal treatment, brain damage with long-lasting neuro-behavioral damage may occur^48–50^. This is more pronounced with nerve agent, in which the preliminary cholinergic hyperstimulation is followed by the activation of other neurotransmitter systems, with the glutamatergic system being the most pronounced^14,18,50–52^. When exposed to moderate or high doses of OPs, casualties may develop long-lasting seizures^34,50^, and if treatment is delayed or absent, seizures could progress to status epilepticus and cause irreversible seizure-related brain damage^51–53^. The typical neuropathological damage was described as bilaterally symmetrical, with severe damage to the temporal lobe structures (i.e., hippocampus, piriform and entorhinal cortices, and amygdala) and the thalamus^52,54–59^. The prevention of seizures following OP poisoning are regarded as a primary objective of medical treatment^34,53^. However, this objective becomes increasingly difficult to achieve as time elapses before therapy is started, and seizures become less responsive to medical treatment^34,52,60^.

According to the Israeli treatment doctrine of nerve agent poisoning, midazolam and Benactyzine should be co-administered for improved therapeutic effect in severe nerve agent poisoning. However, as this combination has the potential to induce side effects, we aim to test novel combinations that will provide as good survival results as this combination, with fewer side effects.

Though benzodiazepine treatment shows that increasing synaptic GABAergic activity is useful, that is not the same as increasing GABA concentrations, especially as GABA itself is subject to dynamic regulation, being formed and degraded continuously.

However, specifically with OPs, it has been reported that sarin directly reacts with muscarinic acetyl-choline receptors present in presynaptic GABAergic neurons in rat hippocampal slices, thereby reducing GABA release^61^. Thus, it was postulated that increasing the concentration of GABA in the brain might have positive effects in OP poisoning. Tiagabine is an anticonvulsant drug that directly inhibits the re-uptake of GABA into presynaptic nerve terminals and increases its concentrations, thus enhancing GABAergic transmission. Despite these features, tiagabine was found ineffective in blocking soman-induced convulsions^62,63^. Vigabatrin is an antiepileptic agent that prevents the metabolism of GABA by irreversibly inhibiting GABA transaminase. This results in increased circulating GABA concentrations by reversing the direction of the transport. As vigabatrin is an irreversible inhibitor of GABA transaminase, its duration of effect is thought to be dependent on the rate of GABA transaminase re-synthesis rather than on the rate of drug elimination. Using vigabatrin alone and in combination with atropine in a mouse model of OP poisoning has shown to protect mice against dimethoate lethality, helped to maintain the righting reflex, and delayed the onset of muscle fasciculations^64^.

Gabactyzine is expected to be able to cross the BBB due to the lipophilic character of its benactyzine component, helping to transport GABA into the brain. Next, it is expected to undergo hydrolysis, releasing concomitantly benactyzine and GABA, which are expected to act synergistically in reducing brain damage following exposure to nerve agents. Moreover, and as centrally-acting drugs have various side effects, we anticipate that by combining the two drugs it could potentially lead to reduction of the total dose of benactyzine being used, thus reducing its side effects while preserving its therapeutic value.

Diffusion-weighted magnetic resonance imaging (DWMRI) allows noninvasive description of biological tissues based on their water diffusion characteristics, and at an early stage. It was already shown that nonspecific brain damage can be depicted early using DWMRI, days and even weeks before it can be detected in T1 and T2 weighted MR images^65,66^. Such an accurate, noninvasive and early assessment of brain tissue response is essential when assessing novel therapeutics against acute poisoning, enabling real time adjustments of treatment. The disadvantage is that the DWMRI is sensitive mainly to early changes in tissue characteristics and less to subtle changes in blood vessels' function which may cause later effects.

In a previous study we have already shown DWMRI as a valid method for monitoring paraoxon-induced brain damage in a rat model, as well as using it for the assessment of early vs late treatment, using two known compounds with beneficial CNS effects^35^. The images in this study were acquired on a 3.0 T scanner in small animals using echo-planar based DWMRI sequences. We have included a LSDI sequence, enabling robust yet low-resolution DWMR images. For this reason, the images are of low resolution, appearing blur. Still, the damage induced by paraoxon is clearly depicted and quantification (ROI analysis), showing significant effects. As we shown previously^35^ and in this study, the early paraoxon-induced toxicity detected in DWMRI precedes later brain atrophy, reflected by the enlarged ventricles.

A successful synthesis was developed for the preparation of gabactyzine **1**. The preliminary animal studies showed promising results regarding the safety of gabactyzine **1**. In the second study we showed preliminary clinical efficacy in OP-poisoned animals, in two administration schedules: early (1 min after seizure initiation) vs. late (30 min). This led us to further investigate the efficacy of gabactyzine **1** in comparison to the standard antidotal treatment in Israel using more elaborated tools.

In means of survival, as shown in the Kaplan–Meier curves of the different treatment groups, early administration of gabactyzine and benactyzine had a similar effect to late administration of midazolam. When applied 30 min post-seizure onset, gabactyzine was less efficient than benactyzine in terms of survival. Early administration of gabactyzine was as good as early administration of benactyzine in preventing brain damage, as assessed by DWMRI and T2-weighted MRI. The later administration of gabactyzine (30 min) seemed to be less effective than the late administration of midazolam, although still better than no treatment at all. Brain atrophy, calculated from ventricular changes on T2-weighted MRI and from DWMRI, was consistent and showed no difference between the treatments. However, since gabactyzine is a new compound, it may be more effective at different doses. Therefore, to determine if the new drug can obtain higher treatment efficacy, dose-escalating studies should be performed. The fact that early administration of gabactyzine enables as good treatment efficacy as benactyzine is encouraging since gabactyzine is expected to have fewer side effects.

A limitation of this study is the lack of correlation of brain damage with histological analysis. Though a potential advantage of using MRI is to replace the need to sacrifice animals for histology in various time point during the study, MRI—at least in such early go-no go studies—would be ideally correlated with histological analysis, at least following euthanasia.

These studies were all defined as part of a preliminary proof-of-concept effort, to test whether there is merit in gabactyzine as a prodrug. Within the generic process of studying a new therapeutic agent, after showing preliminary safety and efficacy, the next steps include testing the compound in a larger set of animals, including another species, as defined in the FDA's "two animal rule", and testing the new compound within various drug combinations, comparing new combinations with known drug combinations, as it is our experience that sometimes a single drug performs quite differently than when it is co-administered with other drugs.

## Conclusion

In this set of preliminary proof-of-concept studies, we focused on the safety and preliminary efficacy of a novel pro-drug, gabactyzine, intended to protect the CNS from organophosphate poisoning. As the preliminary results were generally regarded as similar to results obtained with higher doses of other drug combinations, this will now set the way to future studies, further looking into the effects of this novel pro-drug, including at higher concentrations and doses and longer follow-up periods.

## Data Availability

The datasets generated during and/or analyzed during the current study are available from the corresponding author on reasonable request.
